# Synthesis of CoMoO_4_ Nanofibers by Electrospinning as Efficient Electrocatalyst for Overall Water Splitting

**DOI:** 10.3390/molecules29010007

**Published:** 2023-12-19

**Authors:** Jiahui Fan, Xin Chang, Lu Li, Mingyi Zhang

**Affiliations:** Key Laboratory for Photonic and Electronic Bandgap Materials, Ministry of Education, School of Physics and Electronic Engineering, Harbin Normal University, Harbin 150025, China; 15636092826@163.com (J.F.); cx20220718@163.com (X.C.)

**Keywords:** CoMoO_4_, OER, HER, CNFs, electrospinning

## Abstract

To improve the traditional energy production and consumption of resources, the acceleration of the development of a clean and green assembly line is highly important. Hydrogen is considered one of the most ideal options. The method of production of hydrogen through water splitting constitutes the most attractive research. We synthesized CoMoO_4_ nanofibers by electrospinning along with post-heat treatment at different temperatures. CoMoO_4_ nanofibers show a superior activity for hydrogen evolution reaction (HER) and only demand an overpotential of 80 mV to achieve a current density of 10 mA cm^–2^. In particular, the CoMoO_4_ catalyst also delivers excellent performances of oxygen evolution reaction (OER) in 1 M KOH, which is a more complicated process that needs extra energy to launch. The CoMoO_4_ nanofibers also showed a superior stability in multiple CV cycles and maintained a catalytic activity for up to 80 h through chronopotentiometry tests. This is attributed mainly to a synergistic interaction between the different metallic elements that caused the activity of CoMoO_4_ beyond single oxides. This approach proved that bimetallic oxides are promising for energy production.

## 1. Introduction

Addressing climate change and reducing carbon emissions is conducive to promoting the green transformation of the economic structure, accelerating the production and development of green energy, mitigating the adverse effects of climate change, and reducing the losses caused to the economy and society [1,2,3]. As the foundation of hydrogen energy, electrocatalytic water splitting, which is involved in its production and utilization, has naturally become the focus of attention. For cathodic hydrogen evolution reactions, the notable material (such as Pt-based ones) catalysts are confirmed to have an excellent performance [4,5]. On the other hand, the huge potential created by the OER is what affects the production capacity. Noble-metal-based materials (Ru/IrO_2_) still show the highest catalytic activity toward the generation of oxygen [6,7,8]. However, the huge consumption of notable raw materials was blocking the splitting of water. Because of their abundance and excellent performance, transition metal compounds (TMCs) have been intensively studied as bifunctional electrocatalysts, especially co-based materials, such as Co-NRCNTs, Co-NCNT, CoP, Co_2_P, CoP/CNT, Co@N-C, Ni_0_._33_Co_0_._67_S_2_ nanowire, CoO_x_@CN, CoP/rGO-400, CoO/MoOx, etc. [9,10,11,12,13,14,15,16,17,18].

Different electrodes influence the reaction of water splitting, and using an alkaline solution as a condition to evolve energy is the most commercialized and feasible strategy. During the past years, transition-metal-based catalysts, such as nickel, cobalt, iron, copper, and molybdenum, have been shown to possess tunable electronic, morphological, adsorption, and structural properties, making them promising substitutes for noble metal catalysts [19,20]. In particular, metal molybdate compounds have significant desirable properties, such as non-toxicity, a low cost, and good electrochemical activity, and they have been used in some fields [21,22,23]. As shown in past reports, processed CoMoO_4_ obtained by the CoO/MoOx catalyst only needs a low overpotential at a current density of 10 mA cm^−2^. As bifunctional catalysis is important in water splitting, CoMoO_4_ is also considered one of the choices, due to the above merits [24,25]. From a commercialization point of view, developing a cost-efficient strategy synthesis catalyst is imperative [26,27]. Electrospinning is a controllable method and involves the process of forming jets of polymers and dissolvable materials under the action of an electric field and spinning. After heat treatment, the polymer template is sacrificed to obtain a material with a uniform fiber structure; the ordering of the crosslink structure route for charge and gas transportation can enhance the efficiency of water splitting [28,29,30,31].

Based on the above consideration, we synthesized ${\mathrm{CoMoO}}_{4}$ nanofibers by electrospinning along with post-heat treatment. The CMO-650 catalyst has a stable performance through long electrochemical tests, showing a low overpotential of 80 mV to achieve a current density of 10 mA cm^–2^ in HER. Significantly, the catalysis performance of the CMO-650 nanofibers needs an overpotential of 370 mV at 50 mA cm^−2^, which is smaller than other simple oxides. Furthermore, it applies an excellent stability at a current density of 50 mA cm^−2^ on the alkaline electrode for 80 h. This is mainly due to the synergy between the different metal elements, resulting in the activity of CMO exceeding that of single oxides. This work proves that bimetallic oxides show promise in water splitting.

## 2. Results and Discussion

**Figure 1:**
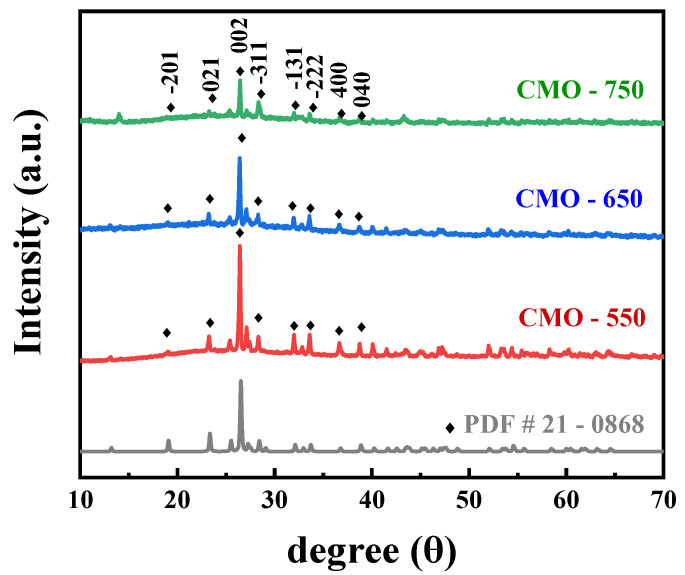
XRD patterns of CMO-550, CMO-650, and CMO-750.

Figure 1 shows the X-ray diffraction (XRD) patterns of the prepared catalysts. The special peaks of CMO-650 can be attributed to the (−201), (021), (002), (−311), (−131), (−222), (400), and (040) phases of CoMoO_4_ (JCPDS No. 21-0868), respectively. The other samples (CMO-550, CMO-750) showed an XRD pattern similar to that of CoMoO_4_ with no peaks. There is a clear tendency for the crystallinity of the materials to decrease, obeying the improved temperature. This phenomenon might be attributed to the grains overlapping with the melting nanofibers and aggregating, causing the unapparent fiber structure under the high treatment.

**Figure 2:**
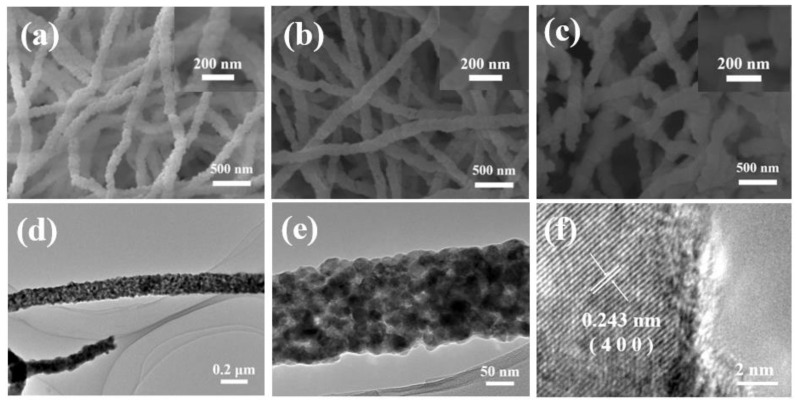
SEM images of (**a**) CMO-550, (**b**) CMO-650, and (**c**) CMO-750. (**d**,**e**) TEM images of CMO-650; (**f**) HR-TEM lattice image of CMO-650.

The morphology and detailed structural information on CoMoO_4_ nanofibers were determined by SEM and TEM (Figure 2a–f). Figure 2a–c show SEM images of CMO-550, CMO-650, and CMO-750. The nanofibers crossed to form a network, which is clearly shown to be distinct, along with boundaries, in Figure 2a,b. The diameter of CMO-650 is around 200 nm. For CMO-750, because the temperature increases in the annealing program, the surface of nanofibers was rougher and the structure of fibers showed a state involving little melting. The CMO-650 nanofibers look like they were obtained through the right condition in order to be an electrode, with the appropriate appearance. For a definitive analysis of the surface and intrinsic composition of the CMO-650, TEM analysis was performed (Figure 2d,f). The sole nanofiber showed a uniform diameter, and the size of the fiber was same as in the shown SEM. A representative high-resolution TEM image is shown in Figure 2f, with the lattice fringe with a distance of around 0.243 nm corresponding to the (400) plane of CoMoO_4_, which confirms the fact that the catalyst synthesized successfully.

**Figure 3:**
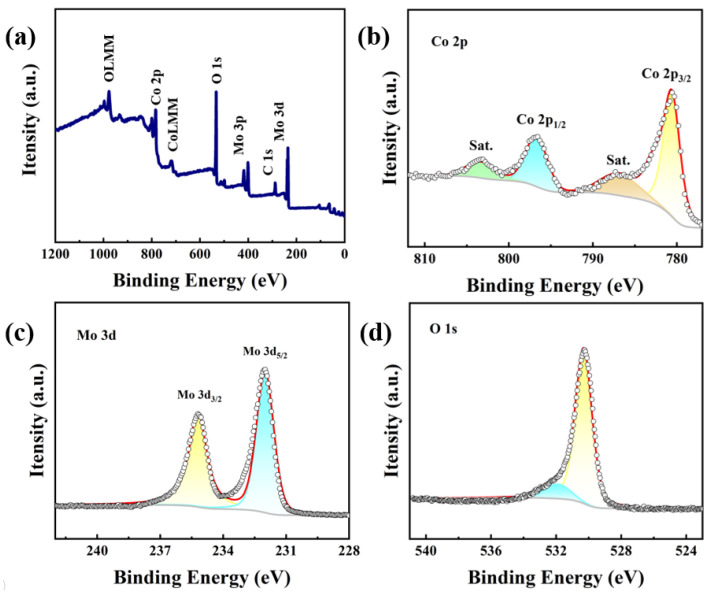
(**a**) XPS survey spectrum for CMO-650. XPS spectra of CMO-650 in the (**b**) Co 2p, (**c**) Mo 3d, and (**d**) O 1s.

The analysis of valence bond changes in the surface components of the material by X-ray photoelectron spectroscopy (XPS) can demonstrate the phase transition process of the materials. As shown in Figure 3a, the peaks of Co, Mo, and O exist in the spectrum for CoMoO_4_. In Figure 3b, the peaks of Co 2p_3/2_ and Co 2p_1/2_ have binding energies of 780.5 and 796.6 eV, which are the characteristics of Co-O species [32,33,34]. The other peaks of Co 2p at 786.8 and 802.8 eV are shake-up satellites. As Figure 3c shows, the binding energies of 232 and 235.2 eV are matched to Mo 3d_5/2_ and Mo 3d_3/2_, which confirms the presence of the Mo VI oxidation state, which is consistent with MoO_4_^2−^. As Figure 3d shows, two peaks with binding energies of 530.3 eV and 531.9 eV are matched to metal-oxygen bonds and OH^-^ groups for O 1s [30,35,36]. These standard characteristic peaks with the right binding energies are deeply obvious, proving that the sample was successfully produced. We also tested the XPS survey spectrum of CMO-550 and CMO-750, and we found that the binding energies of Co and Mo do not have differences. We think that the calcination temperature did not impact the bonding of elements and the values of metals in the test results.

**Figure 4:**
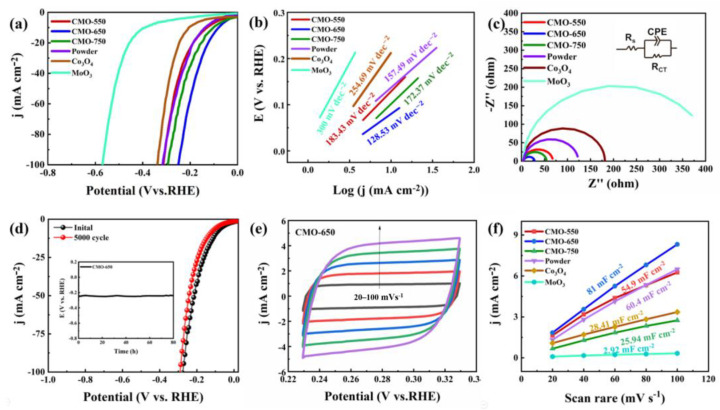
(**a**) LSV curves for CMO-550, CMO-750, CMO-650 powder, Co_3_O_4_, and MoO_3_ for HER with a scan speed of 5 mV s^−1^. (**b**) Tafel slopes for CMO-550, CMO-750, CMO-650 powder, Co_3_O_4_, and MoO_3_. (**c**) EIS pattern of the above catalysts. (**d**) The stability of CMO-650 initially and after 5000 cycles with the inline diagram of the chronopotentiometry test with a current density of 50 mA cm^−2^ for 80 h. (**e**) The different CV curves of CMO-650 ranging from 20–100 mV s^−1^. (**f**) The line fitter between the scan rates and current densities of the above samples.

The performance of the generated hydrogen of CMO-650 (the weight of samples on the carbon paper: 2.5 mg cm^–2^) was examined in 1.0 M KOH. To equally detect CMO-550, CMO-750, CMO-650 powder, Co_3_O_4_, and MoO_3_, all the above catalysts are calculated under the same conditions and possess the same loading on the substrate. In Figure 4a, the CMO-650 electrode reaches a current density of 10 mA cm^−2^ with a low overpotential of 80 mV, which is better than that of the CMO-550 (130 mV), CMO-750 (103 mV), powder (139 mV), CMO-650 powder, Co_3_O_4_ (206 mV) and$\;{\mathrm{MoO}}_{3}$ (390 mV). The results indicate that the Mo oxides have strong synergic effects derived from the introduction of Co elements. The correlation Tafel values are calculated to be 183.43, 128.53, 172.37, 157.49, 254.69, and 300 mV dec^−1^ for CMO-550, CMO-650, CMO-750, CMO-650 powder, Co_3_O_4_, and MoO_3_, respectively (Figure 4b), which means that only a tiny overpotential change is required to meet the rapid increase in current density of CMO-650. Furthermore, electrochemical impedance spectroscopy (EIS) in Figure 4c shows that CMO-650 has the smallest charge transfer resistance (Rct), 17.67 Ω, which is an order of magnitude smaller than CMO-550 (58.7 Ω), CMO-750 (44.544 Ω), CMO-650 powder (113.78 Ω), Co_3_O_4_ (173.575 Ω) and MoO_3_ (403.582 Ω), indicating a fast Faradic process due to the presence of the CMO-650 interface. As shown in Figure 4d, 5000 cycles of CV are performed at a hydrogen evolution potential at a scan rate of 100 mV s^−1^. Testing the polarization curve of the CMO-650 after cycling shows that the potential change is almost acceptable. It is negligible and can be observed by the chronopotentiometry test by applying a current density of 50 mA cm^−2^ on the electrode for 80 h. CMO-650 exhibits an excellent electrochemical stability when tested for stability in alkaline environments. Figure 4e shows the different CV curves of CMO-650 at scan rates ranging from 20 to 100 mV s^–1^, respectively. The corresponding electric double-layer capacitance (C_dl_) value is estimated by linearly fitting the change of current density with the above graph of the corresponding sweep speed. It can be noted from Figure 4f that the best C_dl_ value of CMO-650 is 81 mF cm^–2^. The CMO-550 is 54.9 mF cm^–2^, the CMO-750 is 25.94 mF cm^–2^, the powder is 60.4 mF cm^–2^, the ${\mathrm{Co}}_{3}O_{4}$ is 28.41 mF cm^–2^, and the ${\mathrm{MoO}}_{3}$ is 2.92 mF cm^–2^. This result demonstrates that CMO-650 has a much higher surface area than others, which improves the efficiency of HER.

**Figure 5:**
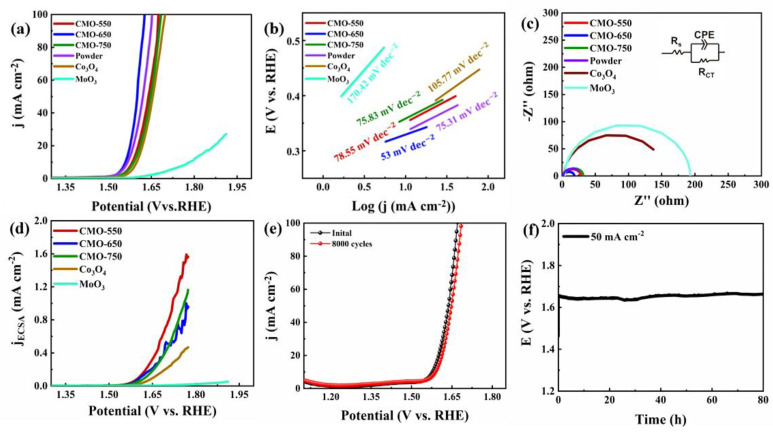
(**a**) LSV curves for CMO-550, CMO-750, CMO-650 powder, Co_3_O_4_, and MoO_3_ for OER with a scan speed of 5 mV s^−1^. (**b**) Tafel plots for CMO-550, CMO-750, CMO-650 powder, Co_3_O_4_ and${\;\mathrm{MoO}}_{3}$. (**c**) EIS pattern of the above catalysts. (**d**) The stability of CMO-650 initially and after 5000 cycles with the inline diagram of the chronopotentiometry test with a current density of 50 mA cm^−2^ for 80 h. (**e**) The different CV curves of CMO-650 ranging from 20–100 mV s^−1^. (**f**) The line fitter between the scan rates and current densities of the above samples.

The CMO-650 catalyst displays an extraordinary performance in the HER test, but the sluggish OER kinetics still hinder commercial applications. Hence, the OER was replaced with the potential range but with the alkaline electrolyte. As Figure 5a shows, the CMO-650 electrode shows a low overpotential at a current density of 50 mA cm^−2^ (370 mV), which is smaller than that of the CMO-550 (410 mV), CMO-750 (416 mV), CMO-650 powder (390 mV), Co_3_O_4_ (423 mV) at 50 mA cm^−2^, and MoO_3_ (668 mV) at 25 mA cm^−2^. CMO-650 reaches a high current density and only needs a small overpotential, which proves that the bimetallic catalyst is better than single oxides. The Tafel slopes are calculated from the correlative LSV results, which are 78.55, 53, 78.53, 75.31, 105.77 and 170.42 mV dec^−1^ for CMO-550, CMO-650, CMO-750 and CMO-650 powder, respectively (Figure 5b). The smallest value of CMO-650 means that the catalyst only needs low energy to offer the current changes. The electrochemical impedance spectroscopy (EIS) renders Nyquist plots, which are fitted with a Randles circuit (Figure 5c). Herein, CMO-650 exhibited the smallest charge transfer resistance (Rct), 12.48 Ω, which is smaller than CMO-550 (21.79 Ω), CMO-750 (25.0214 Ω), CMO-650 powder (25.012 Ω), Co_3_O_4_ (150.14 Ω), and MoO_3_ (184.606 Ω), indicating a fast Faradic process due to the presence of the CMO-650 interface. To separate the surface area effects from the intrinsic activity, Figure 5d shows the ECSA-normalized LSV curves of materials. Interestingly, CMO nanofibers exhibited a higher intrinsic activity than the single oxides. As shown in Figure 5e, the stability of CMO-650 was tested by 8000 CV cycling, and the after-reaction LSV curve was very close to the initial curve, which shows the strong stability of CMO-650. As the chronopotentiometry test of CMO-650 shows, it can be seen that the overpotentials at a current density of 50 mA cm^−2^ do not show significant attenuation after 80 h (Figure 5f).

**Figure 6:**
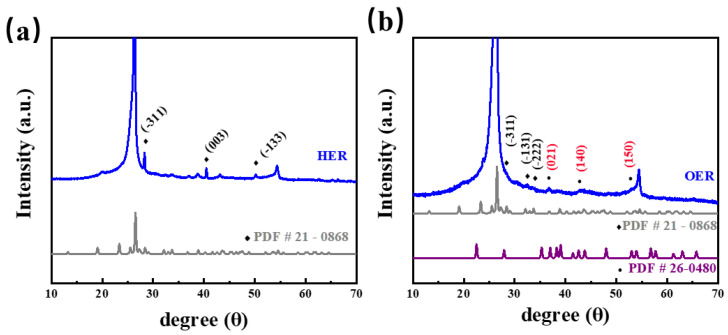
XRD patterns of CMO-650 after stability test; (**a**) after HER and (**b**) after OER.

As shown in Figure 6, CoMoO_4_ remained on both sides of the reaction, which reveals the stability of the catalyst. More interestingly, there were no significant changes in the samples during the HER test, while the CoOOH substance (PDF 26-0480) was synthesized in the Co active site during the OER test. These results indicate that the real active materials resulting from the reconstruction of the bimetallic oxide catalyst and the coupling synergies between different metal elements enhance the electrochemical activity of the catalyst.

## 3. Experimental Section

### 3.1. Materials

Cobalt acetate tetrahydrate (C_4_H_6_CoO_4_·4H_2_O), molybdenylacetylacetonate (C_10_H_14_MoO_6_), potassium hydroxide (KOH), and N-dimethylformamide (DMF) were purchased from Zhiyuan Reagent Corporation (Tianjin, China, Alfa Aesar, Ward Hill, MA, USA). Polyacrylonitrile (PAN) was bought from Sigma-Aldrich Corporation (St. Louis, MO, USA). All reagents were received commercially and used without further purification.

### 3.2. Synthesis of CoMoO_4_ Nanofibers

The electrospinning solution was prepared as follows: 99.63 mg C_4_H_6_CoO_4_·4H_2_O, 130.46 mg C_10_H_14_MoO_6_, and a certain amount of PAN was filled in 5 g DMF. The solution was mixed for almost 24 h until it formed viscous and clear state. We replaced the solution into the electrospin device with the voltage set at 7 kV. After a day, the production was put into muffle, and we calcined the sample at 650 °C for 2 h; the purple nanofibers were named CMO-650. To convert the above sample to the corresponding CMO-X, we changed the relevant temperature (550 °C and 750 °C). For comparison, the CMO-650 powder was synthesized by directly mixing and calcining the above metal salts in muffle at 650 °C.

For comparison, we synthesized Co_3_O_4_ nanofibers using the same method with CMO-650 but not using C_10_H_14_MoO_6_.

### 3.3. Characterizations

The crystal structure of the preparations was determined by X-ray diffraction (XRD) (D/max 2600, Rigaku, Tokyo, Japan). The morphology of the materials was described with a scanning electron microscope (SEM, SU70, Hitachi, Tokyo, Japan). The atomic structure of the catalysts was observed with a transmission electron microscope (TEM, FEI, Tecnai TF20). The surface chemical qualities of the composites were measured by X-ray photoelectron spectroscopy (XPS, Thermofisher Scientific, Waltham, MA, USA).

### 3.4. Electrochemical Measurements

The electrochemical performance of the electrochemical workstation (VMP3): The three-electrode was fabricated using catalysts as the working electrode, and the carbon rod and Ag/AgCl were employed as the counter electrode and reference electrode, respectively. For the working electrode preparation, the catalyst (8.0 mg) and carbon black (acetylene black, 1.0 mg) were mixed in 160 μL of a 5 wt % PVDF solution under ultrasonication for 30 min. After that, apply the above mixture to the carbon paper (evenly coat 20 μL of mixture on carbon paper). All the catalysts were activated by a 50-fold CV test from 0–0.8 V with a scan rate of 50 mV s^−1^. The linear sweep voltammetry (LSV) was conducted from 0 to 0.6 V (vs. Ag/AgCl) at 5 mV s^−1^ in 1 M KOH (pH = 14). For HER, the potential ranged from 0 to −1 V (vs. Ag/AgCl) at 5 mV s^−1^.The LSV curves, corresponding Tafel slopes, chronopotentiometric tests, and cyclic voltammetry were obtained with iR compensation. We use the Single EIS frequency method of 100 kHz to auto iR compensation 85%. An electrochemical impedance spectroscopy (EIS) measurement was conducted at a frequency ranging from 100 kHz to 0.01 Hz at 0.5 V and −1.015 V (vs. Ag/Agcl) for HER and OER, respectively. To measure the electrochemical surface area (ECSA) of all the samples, the C_dl_ was calculated according to the cyclic voltammogram curves with different scan rates.

## 4. Conclusions

In summary, we used electrospun fiber felt as a precursor and selective calcination in air, and different nanofibers were constructed. The HER reaction was tested in an alkaline environment, and the CMO-650 showed a good activity and stability at a current density of 10 mA cm^–2^. Additionally, we found a good electrochemical performance in the OER test because the one-dimensional structure of CMO-650 can effectively combine electrolytes for a rapid mass transfer. The long OER test for 50 mA cm^–2^ can continue for 80 h. All the electrochemical tests with different oxides confirm that the unique fiber structure and bimetallic synergy are preferred to HER and OER. According to XRD, there is almost no change in the surface of the material after the HER reaction, but the material that has undergone water oxidation exhibits a new substance, which is the active substance that truly provides the activity. This work establishes an actionable strategy to provide one-dimensional CMO-650 materials to be used in bifunctional electrochemical catalysis.

## Data Availability

The data presented in this study are available in article.
